# PSC‐DM: A Validated Clinical Diagnostic Model for Distinguishing Primary From Secondary Sclerosing Cholangitis

**DOI:** 10.1111/liv.70892

**Published:** 2026-09-30

**Authors:** Miki Scaravaglio, Rodrigo V. Motta, Laura Cristoferi, Cesare Maino, Alberto Marini, Eugenia Nofit, Eugenia Pesatori, Camilla Gallo, Francesca Bolis, Daphne D'Amato, Armando Curto, Federica Malinverno, Alessio Gerussi, Raffaella Viganò, Mauro Viganò, Stephen J. Rossi, Kris V. Kowdley, Pietro Invernizzi, Alessandra Nardi, Marco Carbone, Emma L. Culver

**Affiliations:** ^1^ Department of Medicine and Surgery University of Milano‐Bicocca Milan Italy; ^2^ Cambridge Stem Cell Institute University of Cambridge Cambridge UK; ^3^ Translational Gastroenterology and Liver Unit, John Radcliffe Hospital, and Nuffield Department of Medicine University of Oxford Oxford UK; ^4^ Division of Gastroenterology Center for Autoimmune Liver Diseases, IRCCS Fondazione San Gerardo Dei Tintori, ERN‐RARE LIVER ‐ Monza Monza Italy; ^5^ Department of Diagnostic Radiology Fondazione IRCCS San Gerardo Dei Tintori Monza Italy; ^6^ Hepatology Unit ASST Papa Giovanni XXIII Bergamo Italy; ^7^ Dipartimento di Medicina Sperimentale e Clinica University of Florence Firenze Italy; ^8^ Hepatology and Gastroenterology Unit ASST Grande Ospedale Metropolitano Niguarda Milan Italy; ^9^ PSC Partners Seeking a Cure Greenwood Village Colorado USA; ^10^ Liver Institute Northwest and Elson S. Floyd College of Medicine Washington State University Seattle Washington USA; ^11^ Department of Mathematics University of Rome Tor Vergata Rome Italy; ^12^ National Institute of Health Research (NIHR) Oxford Biomedical Research Center (BRC), University of Oxford Oxford UK

**Keywords:** autoimmune, differential diagnosis, inflammatory bowel disease, primary sclerosing cholangitis, secondary sclerosing cholangitis

## Abstract

**Background and Aims:**

Primary sclerosing cholangitis (PSC) is a rare, progressive cholangiopathy for which diagnosis remains challenging because of the absence of disease‐specific markers and the presence of secondary causes of sclerosing cholangitis (SSC) that closely mimic its clinical presentation. Diagnostic uncertainty can delay appropriate management and add to the psychological burden experienced by patients. This study aimed to identify clinical parameters that support the early and accurate differentiation of PSC from SSC.

**Methods:**

We conducted a multicentre retrospective study of adults with an established diagnosis of PSC or SSC. Independent predictors of PSC were identified using multivariable logistic regression and externally validated in an independent cohort.

**Results:**

In the derivation cohort (*n* = 234, 155 PSC, 79 SSC), six variables were independently associated with PSC diagnosis, including younger age at presentation (*p* < 0.0001), presence of IBD (*p* < 0.0001), absence of prior hepato‐pancreato‐biliary surgery (*p* < 0.0001), autoimmune comorbidities (*p* = 0.0038), family history of autoimmune diseases (*p* = 0.0086), and absence of pancreatic abnormalities (*p* = 0.0338). These variables were used to develop the PSC diagnostic model (PSC‐DM). In the external validation cohort (*n* = 142, 98 PSC, 44 SSC), the AUROC of PSC‐DM was 0.95 (95% CI 0.91–0.98). In an exploratory analysis, PSC‐DM significantly improved non‐expert diagnostic accuracy (94.3% versus 78.6%, *p* = 0.0023).

**Conclusions:**

PSC‐DM is a simple, externally validated clinical prediction model that accurately distinguishes PSC from SSC using routinely available clinical variables. It may support initial diagnostic assessment in non‐specialist settings, facilitate timely referral to expert centres and improve patient selection for clinical trials.

## Key Points

- Distinguishing primary sclerosing cholangitis (PSC) from secondary sclerosing cholangitis (SSC) remains challenging in the absence of disease‐specific diagnostic markers.
- PSC‐DM is a simple model based on six routinely available clinical variables that estimates the probability of PSC in adults with sclerosing cholangitis.
- PSC‐DM showed good diagnostic accuracy across derivation and external validation cohorts with different SSC aetiologies and outperformed non‐expert clinical assessment in an exploratory comparison.
- PSC‐DM may support initial diagnostic assessment and earlier specialist referral, while atypical or discordant cases require expert evaluation.

## Lay Summary

- Primary sclerosing cholangitis (PSC) is a rare immune‐mediated disease that affects the bile ducts and liver. It can be difficult to diagnose particularly for non‐specialists, because it is uncommon, the initial symptoms often resemble those of other conditions and there is no single test that can reliably confirm the diagnosis. These challenges can lead to significant delays in diagnosis, causing uncertainty and psychological distress for patients and reducing opportunities for early specialist care.
- The PSC Diagnostic Model (PSC‐DM) helps address the diagnostic challenge of PSC using six routinely available clinical features to accurately distinguish PSC from other causes of sclerosing cholangitis. By supporting earlier and more accurate diagnosis, PSC‐DM may help patients access appropriate specialist care sooner and improve consistency in the selection of participants for PSC research studies and clinical trials.

**Abbreviations**
ABCB4: ATP‐binding cassette sub‐family B member 4
AIH: autoimmune hepatitis
AUROC: area under the receiver operating characteristic curve
CI: confidence interval
CT: computed tomography
ERCP: endoscopic retrograde cholangiopancreatography
IBD: inflammatory bowel disease
IgG4: Immunoglobulin G4
IQR: interquartile range
IRC: IgG4‐related cholangitis
MRCP: magnetic resonance cholangiopancreatography
MRI: magnetic resonance imaging
PSC: primary sclerosing cholangitis
PSC‐AIH: PSC‐autoimmune hepatitis variant syndrome
SSC: secondary sclerosing cholangitis
VCTE: vibration‐controlled transient elastography

## 1 Introduction

Primary sclerosing cholangitis (PSC) is a rare, progressive cholangiopathy characterised by fibro‐inflammatory injury of the intra‐ and extra‐hepatic bile ducts that can lead to the development of multifocal strictures, chronic cholestasis, liver failure and increased risk of hepatobiliary malignancies [1, 2, 3, 4, 5]. Early identification is fundamental to ensure timely referral to specialist centres and cancer surveillance [5]. Current international guidelines provide criteria for establishing a diagnosis of PSC based on characteristic cholangiographic or histological features of sclerosing cholangitis, combined with chronic elevation of cholestatic liver enzymes and frequently coexisting inflammatory bowel disease (IBD) [1, 2, 6]. However, in the absence of pathognomonic features of PSC, the diagnosis is often uncertain. Consequently, diagnostic assessment still relies heavily on expert interpretation, contributing to variability across centres and delaying recognition in non‐specialist settings.

A diagnosis of PSC requires exclusion of identifiable causes of secondary sclerosing cholangitis (SSC) [6]. SSC comprises a heterogeneous group of rare disorders that result from a known pathological process or injury, including surgical or traumatic biliary injury, ischaemic cholangiopathy, chronic pancreatitis, infectious or toxic insults, congenital disorders and immunoglobulin G4 (IgG4)‐related cholangitis (IRC). The epidemiology of SSC remains poorly defined because of its diverse aetiologies and the absence of dedicated population‐based studies [7, 8]. The clinical and radiological presentation of SSC frequently overlaps with that of PSC, particularly in IRC, which is the biliary manifestation of IgG4 related disease [9, 10]. As no disease‐specific diagnostic marker is currently available [11], distinguishing PSC from SSC remains a major challenge in clinical practice.

Diagnostic ambiguity between PSC and SSC impacts patient management. PSC carries a poor prognosis with progression to liver failure and increased risk of biliary and colorectal malignancies that requires lifelong surveillance [12, 13, 14, 15, 16, 17, 18]. In contrast, the natural history of SSC varies considerably according to the underlying aetiology. Some forms stabilise or improve following treatment; others progress to liver failure and transplantation [7, 10]. Whilst no disease‐modifying therapy is currently approved for PSC, many forms of SSC may respond to targeted treatment if recognised early [7, 19]. Furthermore, delayed or incorrect diagnosis contributes to significant psychological distress in patients [15]. More accurate disease classification may also be relevant to clinical trials. Although SSC is typically excluded from PSC clinical studies [20], the absence of standardised criteria to differentiate PSC from SSC may contribute to diagnostic heterogeneity across trial populations [21].

In this multicentre study, we aimed to develop and externally validate a predictive model to distinguish PSC from SSC using routinely available clinical parameters. We further compared its diagnostic performance with that of non‐expert clinicians to assess its potential utility in routine clinical practice.

## 2 Methods

### 2.1 Study Design and Participants

This multicentre, retrospective cohort study included patients diagnosed with sclerosing cholangitis between January 1995 and June 2023, with follow‐up data available until June 2024. The derivation cohort included patients followed up at three liver centres in Italy, Fondazione IRCCS San Gerardo dei Tintori (Monza, Italy), ASST Papa Giovanni XXIII (Bergamo, Italy), ASST Grande Ospedale Metropolitano Niguarda (Milan, Italy). External validation was performed using data from the Oxford University Hospitals NHS Foundation Trust (Oxford, UK). To minimise selection bias, all individuals with at least one hospital encounter for clinical assessment, liver biopsy, magnetic resonance cholangiography (MRCP) or endoscopic retrograde cholangiography (ERCP) were identified through electronic health record review using predefined keywords: ‘sclerosing cholangitis’, ‘primary sclerosing cholangitis’, ‘PSC’, ‘secondary sclerosing cholangitis’, ‘SSC’, ‘biliary stricture’, ‘periductal fibrosis’, ‘peribiliary fibrosis’. Adults with a confirmed diagnosis of sclerosing cholangitis and at least 1 year of follow‐up were included. Exclusion criteria were less than 1 year of follow‐up, prior liver transplantation or presentation with decompensated advanced chronic liver disease precluding reliable aetiological assessment. Patients with small duct PSC and variant syndrome with autoimmune hepatitis (PSC‐AIH) [22, 23], were included within the PSC spectrum. Sclerosing cholangitis was defined by strictures in intra‐ and/or extrahepatic bile ducts on MRCP or ERCP and/or periductal fibrosis on liver biopsy [1]. PSC was diagnosed according to international guidelines, based on characteristic cholangiographic and/or histological findings after exclusion of secondary causes of sclerosing cholangitis [1, 6]. Cases with an identifiable aetiology were classified as SSC. IRC was diagnosed according to the HISORt criteria [10]. Detailed diagnostic definitions are provided in Supporting information S1. Indeterminate cases were centrally reviewed by a panel of three experts from the participating centres.

### 2.2 Variables of Interest

Data was collected at diagnosis or first specialist consultation. Variables of interest included age at PSC or SSC diagnosis, sex, presence of IBD, prior hepato‐pancreato‐biliary surgery, autoimmune comorbidities, family history of autoimmunity in first‐degree relatives, family history of gallstones or cholecystectomy before the age of 40, evidence of pancreatic abnormalities on magnetic resonance imaging (MRI) or computed tomography (CT), concomitant liver diseases, total immunoglobulins and IgG4, advanced chronic liver disease and genetic testing results when available. IBD was defined by consistent endoscopic and/or histological findings. Pancreatic abnormalities were defined as radiological features suggestive of autoimmune pancreatitis or acute or chronic pancreatitis. Features suggestive of autoimmune pancreatitis included diffuse or focal pancreatic enlargement and a peripancreatic capsule‐like rim. Features suggestive of acute or chronic pancreatitis included pancreatic atrophy, calcifications, irregular dilatation of the main pancreatic duct or side branches, pancreatic duct strictures, pancreatic cysts and intraductal calculi [7, 10]. Advanced chronic liver disease was defined histologically or by liver stiffness ≥ 9.6 kPa on vibration‐controlled transient elastography (VCTE) [24].

### 2.3 Statistical Analysis

Continuous variables were reported as median and interquartile range (IQR), categorical variables as absolute numbers and percentages. For group comparisons, we used the *χ* ^2^ test for categorical variables (or Fisher's exact test in the case of sparse tables or MCNemar test for paired observations) and Student's *t*‐test for continuous variables (or Wilcoxon rank‐sum test when a significant departure from normality was detected). Candidate variables for model development were selected a priori based on clinical relevance and their potential role in the differential diagnosis between PSC and SSC. These included age at PSC or SSC diagnosis, presence of IBD, previous abdominal surgery, other autoimmune comorbidities, family history of autoimmunity, family history of gallstones or cholecystectomy before the age of 40, concomitant liver diseases, radiological pancreatic abnormalities, total immunoglobulins and serum IgG4. The logistic model was used to derive the final model. Variables were selected through non‐automated backward selection, considering correlation structure among covariates and clinical interpretation of their effects. A linear effect was assumed for continuous variables and validated using smoothed plots of deviance residuals versus covariate values. Fractional polynomials transformations were also considered. Due to quasi‐complete separation of data, Firth's penalised maximum likelihood estimation was applied to reduce bias in parameter estimates [25]. Significance was assessed using Likelihood Ratio test statistic. Poorly predicted observations were identified by standardised deviance residuals. Model performance was evaluated using calibration curves and AUROC. AUROC confidence bands were computed by non‐parametric stratified bootstrapping. Calibration curves were smoothed using generalised additive model. Analyses were performed using SAS (version 9.4) and R (version 4.4.1).

### 2.4 Ethics Statement

The study was conducted in accordance with the Declaration of Helsinki and approved by local ethics committees (University of Milano‐Bicocca: N° 4483_09012024; University of Oxford: N° 10/H064/51, N° 21/YH/0206).

### 2.5 Patient and Public Involvement

The study proposal was presented to PSC Partners Seeking a Cure, an international patient organisation dedicated to advancing PSC research and supporting individuals affected by the disease. This provided the opportunity to incorporate patient perspectives into the study and to support dissemination of the findings to the broader PSC community.

## 3 Results

### 3.1 Derivation Cohort

Of 308 screened cases, 234 met inclusion criteria, comprising 155 PSC and 79 SSC patients (Figure S1). Median age at diagnosis was 33.1 years (IQR 22.5–46.5) for PSC and 68.5 years (IQR 57.9–76.3) for SSC. Both groups showed male predominance (PSC 58.7%, SSC 62.0%) and a high prevalence of large duct disease (PSC 90.3%, SSC 98.7%). Among SSC patients, the leading aetiology was choledocholithiasis (55 cases, 69.6%), followed by traumatic or ischaemic biliary injury (16 cases, 20.2%) (Table 1). Nineteen patients (12.3%) had PSC‐AIH variant syndrome confirmed on liver biopsy. Cholangiography was available for all patients. MRCP was performed in all patients, except two SSC patients assessed by ERCP and CT. Typical cholangiographic features of PSC with multiple strictures and dilations in both intrahepatic and extrahepatic biliary tree [26] were identified in 80.7% of large duct PSC and 18.0% of large duct SSC patients. The remaining patients in both groups had isolated intrahepatic or extrahepatic strictures. Small duct disease was identified in 15 PSC (9.7%) and one SSC patient (1.3%). Two thirds of small duct PSC had concomitant IBD; the remainder were diagnosed with PSC after exclusion of genetic cholestasis. In the overall cohort, IBD was present in 60.6% of PSC and absent in SSC. Autoimmune comorbidities and family history of autoimmunity were more frequent in PSC (26.5% and 27.5%) than in SSC (3.8% and 5.1%). Prior hepato‐pancreato‐biliary surgery was reported in a total of 20 (12.9%) PSC and 51 (64.6%) SSC patients. All 20 PSC patients underwent previous cholecystectomy. Among the 51 SSC patients, 47 underwent cholecystectomy and the others pancreaticoduodenectomy (Whipple procedure). Pancreatic abnormalities were observed in 1.3% of PSC and 32.9% of SSC patients (Table S1).

**TABLE 1:** Characteristics of the derivation cohort.

	Derivation cohort (*n* = 234)	
	PSC (*n* = 155)	SSC (*n* = 79)
Age, years	33.1 (22.5, 46.5)	68.5 (57.9, 76.3)
Sex, male	91 (58.7)	49 (62.0)
Type of disease		
LD	140 (90.3)	78 (98.7)
SD	15 (9.7)	1 (1.3)
Aetiology		
Choledocholithiasis	—	55 (69.6)
Traumatic or ischaemic biliary injury	—	16 (20.2)
IRC	—	7 (8.9)
Genetic	—	1 (1.3)
IBD	94 (60.6)	0 (0.0)
Previous biliary surgery	20 (12.9)	51 (64.6)
Autoimmune comorbidities	41 (26.5)	3 (3.8)
Familial autoimmunity	42 (27.5)	4 (5.1)
Pancreas abnormalities	2 (1.3)	26 (32.9)
ACLD	25 (16.1)	15 (19.0)

*Note:* Data are median (IQR) or *n* (%). Dashes (—) indicate not applicable (aetiology recorded for SSC only).

Abbreviations: ACLD, advanced chronic liver disease; IBD, inflammatory bowel disease; IRC, IgG4‐related cholangitis; LD, large duct; PSC, primary sclerosing cholangitis; SD, small duct; SSC, secondary sclerosing cholangitis.

### 3.2 Multivariable Analysis

Logistic regression was used to derive the best‐fitting model to predict PSC diagnosis. Six independent predictors of PSC were identified, including younger age at diagnosis (AgeDiag, *p* < 0.0001), presence of IBD (Ibd, *p* < 0.0001), absence of previous hepato‐pancreato‐biliary surgery (PreSurg, *p* < 0.0001), non‐IBD autoimmune comorbidities (OthAut, *p* = 0.0038), family history of autoimmune diseases (FamAut, *p* = 0.0086) and absence of pancreatic abnormalities (Pancreas, *p* = 0.0338) (Table 2). The absence of pancreatic abnormalities remained significant even after exclusion of IRC cases (Table S2).

**TABLE 2:** Estimated parameters for PSC diagnosis in the model derivation cohort.

	Parameter estimate	Standard error	LR Chi‐square	*p*
Intercept	6.6417	1.4139		
Age (years)	−0.1212	0.0262	40.8946	< 0.0001
IBD	4.9673	1.4440	33.3003	< 0.0001
Previous biliary surgery	−2.5868	0.6432	18.5523	< 0.0001
Autoimmune comorbidities	2.2197	0.8064	8.3760	0.0038
Familial autoimmunity	2.3572	0.8880	6.9118	0.0086
Pancreas abnormalities	−2.5001	1.2220	4.5054	0.0338

*Note:* The results are from the logistic model based on baseline characteristics. Derivation cohort *n* = 234, used observations *n* = 232.

Abbreviations: IBD, inflammatory bowel disease; LR, likelihood ratio.

### 3.3 Development of the Score

Using the estimated parameters of the selected variables (Table 2), we developed a score to predict PSC diagnosis:

$$\begin{array}{c} \mathrm{PSC}‐\mathrm{DM}=6.6417–0.1212\times \text{(AgeDiag)}+4.9673\times \text{(Ibd)}\\ \;-2.5868\times \text{(PreSurg)}+2.2197\times \text{(OthAut)}\\ \;+2.3572\times \text{(FamAut)}-2.5001\times \text{(Pancreas)}. \end{array}$$

Based on PSC‐DM, the predicted probability of PSC diagnosis was estimated as follows:

$$\begin{array}{c} \text{Probability}\;\left(\mathrm{PSC}\;\text{diagnosis}\right)=\mathrm{Exp}\left(\mathrm{PSC}‐\mathrm{DM}\right)/\left(1+\mathrm{Exp}\left(\mathrm{PSC}‐\mathrm{DM}\right)\right). \end{array}$$

Internal validation of PSC‐DM showed high discrimination (AUROC 0.989, 95% CI 0.980–0.998; Figure 1A) and calibration (Figure 1B), with clear separation of predicted probabilities between PSC and SSC (Figure 1C,D). The discriminatory performance of the model remained good even when the analysis was restricted to PSC patients without IBD (AUROC 0.97, 95% CI 0.95–0.99) (Figure S2). Analysis of residuals identified three (1.3%) poorly predicted observations. One PSC patient diagnosed at age 63 without IBD but large duct progressive disease and autoimmune thyroid disease was misclassified with a low predicted probability of PSC (predicted probability, 0.15). Two patients with low phospholipid associated cholelithiasis (LPAC) syndrome, diagnosed at ages 19 and 34, the latter carrier of a pathogenic *ABCB4* variant, were misclassified with high predicted probabilities of PSC diagnosis (predicted probabilities ≥ 0.85). A calculator based on PSC‐DM is available online (psc‐dm‐calculator).

**FIGURE 1:**
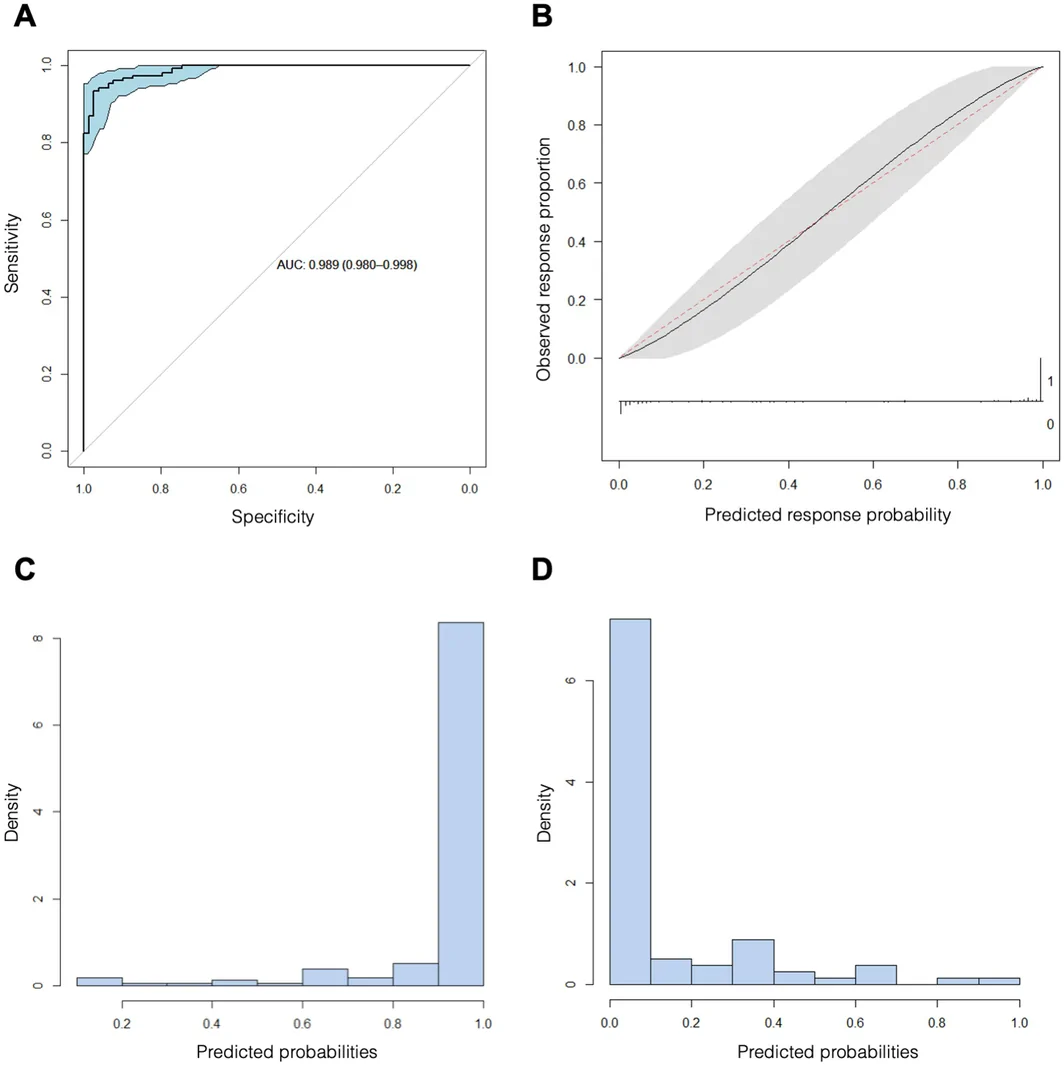
Performance of the model in the derivation cohort. (A) AUROC for the prediction of PSC diagnosis calculated by PSC‐DM in the derivation cohort. (B) Predicted versus observed probability of PSC diagnosis in the derivation cohort. (C) Distribution of the predicted probabilities of PSC diagnosis in the observed PSC and (D) observed SSC in the derivation cohort. Derivation cohort *n* = 234, used observations *n* = 232. PSC, primary sclerosing cholangitis; SSC, secondary sclerosing cholangitis.

### 3.4 Validation Cohort

External validation included 142 patients with sclerosing cholangitis (98 PSC, 44 SSC) followed at the Oxford University Hospitals NHS Foundation Trust, Oxford (UK) in the same time frame as the derivation cohort. Median age at diagnosis was 32.9 years (IQR 22.2–46.7) for PSC and 64.1 years (IQR 47.6–73.8) for SSC. The predominant SSC aetiology was IRC (35 cases, 79.6%), followed by choledocholithiasis (4 cases, 9.1%) (Table 3). Large duct disease was reported in 73.5% of PSC and 97.7% of SSC. IBD was present in 79.6% of PSC and 2.3% of SSC. Of 26 small duct PSC cases, 24 (92.3%) had concomitant IBD, while in the remaining two, genetic cholestasis was excluded before confirming the PSC diagnosis. No PSC‐AIH variant syndrome was included.

**TABLE 3:** Characteristics of the validation cohort.

	Validation cohort (*n* = 142)	
	PSC (*n* = 98)	SSC (*n* = 44)
Age, years	32.9 (22.2–46.7)	64.1 (47.6, 73.8)
Sex, male	52 (53.1)	30 (68.2)
Type of disease		
LD	72 (73.5)	43 (97.7)
SD	26 (26.5)	1 (2.3)
Aetiology		
Choledocholithiasis	—	4 (9.1)
Traumatic or ischaemic biliary injury	—	2 (4.5)
IRC	—	35 (79.6)
Genetic	—	1 (2.3)
Ketamine induced SC	—	2 (4.5)
IBD (%)	78 (79.6)	1 (2.3)
Previous biliary surgery	9 (9.2)	8 (18.2)
Autoimmune comorbidities	13 (13.3)	4 (9.1)
Familial autoimmunity	11 (12.4)	3 (7.3)
Missing data	9 (9.2)	3 (6.8)
Pancreas abnormalities	1 (1.0)	32 (72.7)
ACLD	4 (4.1)	2 (4.7)

*Note:* Data are median (IQR) or *n* (%). Dashes (—) indicate not applicable (aetiology recorded for SSC only).

Abbreviations: ACLD, advanced chronic liver disease; IBD, inflammatory bowel disease; IRC, IgG4‐related cholangitis; LD, large duct; PSC, primary sclerosing cholangitis; SC, sclerosing cholangitis; SD, small duct; SSC, secondary sclerosing cholangitis.

PSC‐DM maintained strong discriminatory performance (AUROC 0.945, 95% CI 0.907–0.983; Figure 2A), with clear separation of the predicted probabilities between PSC and SSC (Figure 2C,D). The calibration of the model was satisfactory, with an overestimation tendency for the predicted probabilities at extreme high values (Figure 2B). Defining a probability threshold of 0.5, PSC‐DM achieved a sensitivity of 0.91, a specificity of 0.83 and an overall accuracy of 0.88. The positive predictive value was 0.92, while the negative predictive value was 0.81. Analysis of residuals identified nine (6.3%) poorly predicted observations. Three PSC patients had predicted probabilities of PSC diagnosis below 0.20. These individuals shared atypical features, including older age at diagnosis, absence of IBD, absence of autoimmune comorbidities and family history of autoimmunity. Two had prior hepato‐pancreato‐biliary surgery. In the SSC group, six patients were misclassified with high predicted probabilities of PSC, including two young patients with LPAC syndrome, one of whom carried a pathogenic *ABCB4* variant (predicted probability, > 80%), two young patients with ketamine‐induced SSC (predicted probability, > 95%) and two IRC patients with autoimmune comorbidities, Type 1 diabetes and IBD respectively (predicted probability, > 95%), the latter with histologically‐confirmed IgG4‐autoimmune pancreatitis. When considering only patients with IRC, the predicted probability of PSC was very low in most IRC cases (Figure S3).

**FIGURE 2:**
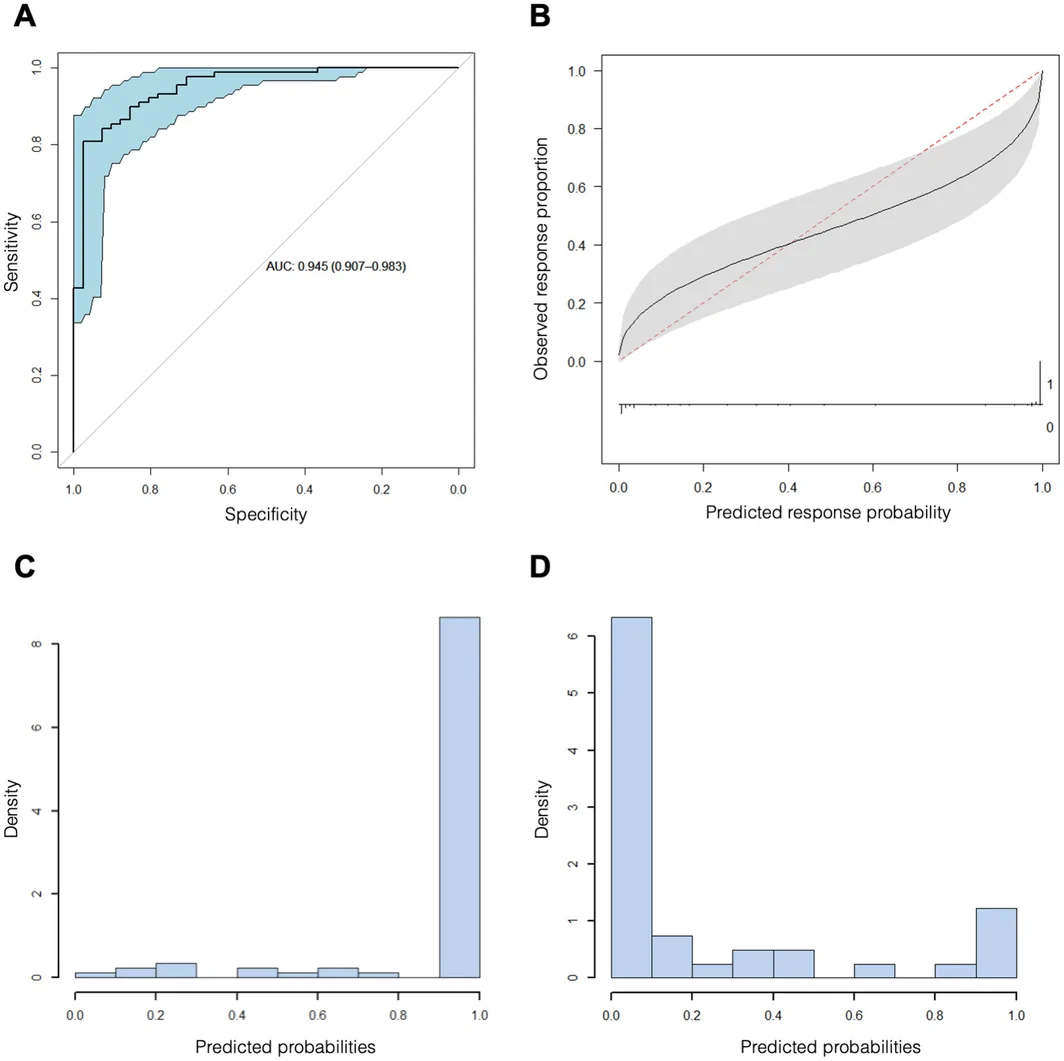
Performance of the model in the validation cohort. (A) AUROC for the prediction of PSC diagnosis calculated by PSC‐DM in the validation cohort. (B) Predicted versus observed probability of PSC diagnosis in the validation cohort. (C) Distribution of the predicted probabilities of PSC diagnosis in the observed PSC and (D) observed SSC in the validation cohort. Validation cohort *n* = 142, used observation *n* = 132. PSC, primary sclerosing cholangitis; SSC, secondary sclerosing cholangitis.

### 3.5 Comparison of PSC‐DM Performance With Non‐Expert Clinical Assessment

As an exploratory analysis, PSC‐DM performance, using a predefined probability threshold of 0.5, was compared with the independent diagnostic assessment of three physicians without specific expertise in PSC in a randomly selected subset of 70 patients. PSC‐DM correctly classified 66/70 cases compared with 55/70 by non‐expert clinicians, demonstrating significantly higher diagnostic accuracy (94.3% versus 78.6%; *p* = 0.0023). Misclassification by non‐experts predominantly occurred in PSC patients without IBD. Notably, PSC‐DM correctly identified 5/6 (83.3.%) of these cases, assigning a probability of PSC greater than 0.85 in all cases. Non‐experts outperformed PSC‐DM in only one SSC case diagnosed with LPAC syndrome at age 19 (Table S3).

## 4 Discussion

This multicentre study presents the development and validation of PSC‐DM, a simple clinical model that accurately distinguishes PSC from SSC using six routinely available clinical variables: age, concomitant IBD, other autoimmune comorbidities, familiarity for autoimmunity, prior hepato‐pancreato‐biliary surgery and pancreatic abnormalities. The model demonstrated good discrimination and calibration in both derivation and validation cohorts and improved non‐expert diagnostic assessment in an exploratory comparison. These findings suggest that PSC‐DM could provide structured support at an early stage of the diagnostic workflow, particularly in non‐specialist settings where differentiation between PSC and SSC may be challenging.

The variables retained in PSC‐DM reflect established clinical differences between PSC and SSC. Young age at presentation and concomitant IBD are well‐recognised features of PSC [4, 7]. Although PSC is commonly considered an autoimmune liver disease, its immunobiology is atypical. No disease‐specific autoantigen or diagnostic autoantibody has been identified and conventional immunosuppression has not demonstrated meaningful clinical benefit [12]. Nevertheless, strong immunogenetic evidence, together with the well‐established association of PSC with IBD and other autoimmune comorbidities, including Type 1 diabetes, coeliac disease, thyroid disease and rheumatoid arthritis [27], support an important role for immune‐mediated mechanisms [5]. Of note, all these autoimmune comorbidities were observed in our cohort. Familial clustering of autoimmunity is well documented in PSC [28] and while some SSC subtypes, notably IRC, share genetic risk loci with other autoimmune diseases [29, 30] and may exhibit familial autoimmune clustering [31], these associations were less prevalent than in PSC. In our cohort, autoimmune comorbidities and family history of autoimmunity occurred in 26.5% and 27.5% of PSC, compared with 3.8% and 5.1% of SSC. Conversely, pancreatic abnormalities and previous biliary surgery are more commonly associated with SSC. Pancreatic abnormalities are classically associated with IRC which presents with Type 1 autoimmune pancreatitis in up to 90% of patients [30] and have been reported also in a minority (7%–15%) of PSC patients [32, 33]. In our cohort, radiological pancreatic abnormalities were substantially more frequent in SSC and were observed across different aetiologies, including choledocholithiasis‐associated SSC and chronic pancreatitis. These findings suggest that pancreatic abnormalities may reflect a broad spectrum of pancreatobiliary disease associated with SSC, thereby providing useful information in the differential diagnosis between PSC and SSC. Previous hepato‐pancreato‐biliary surgery likewise was highly characteristic of SSC, reflecting the value of a detailed surgical history [7]. SSC often arises from surgical complications causing traumatic or ischaemic biliary injury. Patients with biliary lithiasis or genetic cholestasis and recurrent lithiasis frequently undergo cholecystectomy and those with IRC Whipple procedure [10, 34].

The potential clinical value of PSC‐DM lies in addressing the uncertainties of the current diagnostic workflow for PSC. Cholangiographic, histological and biochemical findings establish a diagnosis of sclerosing cholangitis but do not always reliably discriminate PSC from SSC [11, 35, 36]. PSC‐DM complements these investigations by systematically integrating routinely available clinical features that may otherwise be overlooked or given a different weight in the diagnostic assessment, particularly by clinicians less familiar with PSC. This was supported by the exploratory comparison with non‐expert physicians, in which PSC‐DM demonstrated higher overall diagnostic accuracy. Earlier and more accurate differential diagnosis impact clinical management. Correct identification of PSC is important for appropriate hepatobiliary and colorectal cancer surveillance, particularly because around one third of malignancies occur within the first year of disease onset [18, 37, 38, 39, 40]. On the other side, avoiding an incorrect diagnosis of PSC may prevent patients with SSC from undergoing unnecessary and burdensome lifelong surveillance. From a therapeutic perspective, while no disease‐modifying therapy currently exists for PSC, several SSC subtypes are potentially treatable, for instance, immunosuppression or anti‐CD20 agents in IRC [10, 19], ileal bile acid transporter inhibitors in genetic cholestasis [41, 42, 43] and drug discontinuation in ketamine‐induced cholangiopathy [44]. Consequently, PSC‐DM, by prompting consideration of secondary causes in patients with low predicted probability of PSC, may help direct further aetiological investigation and reduce missed opportunities for targeted management. In addition, PSC‐DM may provide a more standardised approach to disease classification in clinical trials. Regulatory bodies mandate exclusion of SSC from PSC clinical trials [20], yet the absence of standardised criteria for differentiating the two conditions may contribute to variability in patient selection across recruiting centres. This is reflected in published clinical trials where the proportion of PSC‐IBD patients ranges from 48% to 89% [21, 45]. In PSC clinical trials, even limited misclassification could influence estimates of treatment effect, either by introducing SSC patients who respond through a different disease mechanism or by diluting a PSC‐specific therapeutic effect (e.g., targeting the gut‐liver axis). Although a reproducible clinical model is unlikely to replace a rigorous trial‐specific diagnostic adjudication, PSC‐DM may provide an additional objective framework to support more consistent patient selection.

A key strength of our study is the external validation of PSC‐DM in a cohort with a substantially different distribution of SSC aetiologies from the derivation cohort. Despite these differences, the model performance remained robust, supporting its generalisability across different sclerosing cholangitis subtypes. The validation cohort was predominantly represented by patients with IRC, a clinically important and challenging differential diagnosis of PSC. Although IRC is a distinct systemic immune‐mediated disease with characteristic clinical, radiological and pathophysiological features [10], its inclusion within the non‐PSC group reflects the intended real‐world application of PSC‐DM. The low predicted probability of PSC in most IRC cases supports the model's ability to distinguish PSC from IRC as well as other forms of SSC. Patients in which the performance of PSC‐DM was less accurate are equally informative, as they highlight clinical scenarios in which additional diagnostic information not captured by the model may help distinguish PSC from its mimickers. The model overestimated the probability of PSC diagnosis in specific SSC subgroups, including LPAC syndrome, IRC with concomitant IBD and ketamine‐induced sclerosing cholangitis. In LPAC syndrome, particular attention should be paid to the pattern of gallstone disease, including symptomatic lithiasis at a young age, recurrence after cholecystectomy, intrahepatic lithiasis. *ABCB4* deficiency is identified in only 30%–50% of cases and its absence therefore does not exclude the diagnosis [34, 46]. As LPAC can closely mimic the radiological and/or histological features of PSC [1, 34], a suggestive clinical phenotype should prompt specialist assessment and consideration for genetic testing, consistent with recommendations for atypical large duct PSC and small duct PSC without IBD [1, 47, 48]. Similarly, concomitant IBD, although strongly associated with PSC, occurs in 5%–10% of IRC cases [10] and may therefore reduce the discriminatory performance of PSC‐DM. In this setting, the diagnosis should not rely on IBD status alone and require specialist assessment. Finally, ketamine‐induced sclerosing cholangitis is increasingly diagnosed among medical and recreational ketamine users [44] and may closely mimic PSC, particularly in young adults. As exposure to ketamine is not captured by PSC‐DM, a careful history of medical and recreational drug use remains essential, especially in the presence of co‐existing uropathy [49, 50]. Conversely, PSC‐DM underestimated the probability of PSC in fewer than 3% of patients, all of whom had atypical presentations, including older age, absence of IBD or autoimmune features and previous hepato‐pancreato‐biliary surgery. Although these cases provide relevant information on potential clinical features that are currently missing in the model, the small number and heterogeneity of these cases precluded further model refinement. Future refinement should be assessed prospectively in larger cohorts including atypical PSC and rare PSC mimickers. Importantly, PSC‐DM is intended to support non‐expert clinicians in the initial diagnostic assessment of sclerosing cholangitis, while atypical and challenging cases should prompt specialist review.

We acknowledge that the study has limitations. Small duct PSC and PSC‐AIH variant syndrome were included to reflect real‐world clinical heterogeneity, but subgroup analyses were limited by sample size. Colonoscopy was systematically performed in PSC, whereas only when clinically indicated in SSC, possibly underestimating IBD prevalence in SSC. Despite serum IgG4 evaluation having been recommended by recent guidelines to distinguish large duct PSC from IRC [1, 51, 52], it could not be included in the model because data were unavailable for most non‐IRC SSC cases. Instead, pancreatic abnormalities on MRI or CT provided a more consistently available and discriminatory feature across SSC subtypes. Although some autoantibodies may hold diagnostic value in PSC [53, 54], they are not currently recommended for diagnostic assessment or risk stratification by international guidelines [1]. Consistently, p‐ANCA did not improve the performance of PSC‐DM in an exploratory analysis. Finally, not all SSC aetiologies were represented.

In conclusion, PSC‐DM is a simple, externally validated clinical prediction model that accurately distinguishes PSC from SSC using routinely available clinical variables. PSC‐DM addresses a preexisting gap by providing a pragmatic tool to support diagnostic assessment in non‐specialist settings, facilitate timely referral to expert centres and potentially improve patient selection for clinical trials. Prospective studies are needed to determine whether diagnostic improvements translate into a measurable benefit in clinical practice.

## Author Contributions

M.C., E.L.C., A.N., M.S., R.V.M., L.C. have participated in the study concept and design. A.N. performed and M.C. and M.S. participated in data analysis. M.S., R.V.M., A.M., C.M., E.N., E.P., C.G., F.B. contributed to recruitment and/or ascertainment of participants. M.S. and R.V.M. have verified the underlying data and wrote the first draft. All authors critically read and contributed to the final version of the manuscript.

## Funding

M.S. acknowledges funding from ESOT and Wellcome Trust Clinical Research Training Fellowship (G343115). R.V.M. has received grant support from PSC Support. M.C. has received grant support from Children PSC Foundation, AIRCS, AMAF, EpaC. E.L.C. has received grant support from NIHR‐BRC Oxford, PSC Support. This research was supported by grant from Ministero dell'Università e della Ricerca‐NextGenerationEU PNRR M4.C2.I1.1‐ Avviso 104/2022, CUP 2022‐NAZ‐0581, PRIN PNRR 2022‐NAZ‐0526, PRIN 2022 PNRR‐ Missione 4, Componente 2, Investimento 1.1—Avviso 1409/22—INNOVAtive tissue and non‐invasive biomarkers for a precision medicine in CHOLANGIOcarcinoma: early diagnosis and therapeutic response of tumour subtypes, 2022‐NAZ‐0199, PNRR per la Missione 4, componente 2 Investimento 1.1‐ Avviso 104/2022; Biomarkers of Inflammaging in Liver Diseases (BILD): new directions for basic research and therapeutic approaches in chronic cholangiopathies and cholangiocarcinoma; PRIN: PROGETTI DI RICERCA DI RILEVANTE INTERESSE NAZIONALE—Bando 2020 Prot. 2020KYHTMA, DEMO: Diseases modelling and early therapeutic tools in chronic cholangiopathies and cholangiocarcinoma.

## Conflicts of Interest

L.C. has received speaker fees from Albireo/IPSEN, Advanz/Intercept and advisory/consultancy fees from Albireo/IPSEN, Mirum, Dr. Falk Pharma, Amgen. A.G. has received lecture fees from Ipsen and Advanz, consulting fees from Ipsen, Gilead, Astrazeneca, Signanthealth, CAMP4 therapeutics, travel grants from Ipsen, Advanz and Zydus, unrestricted grant support from Ipsen and is in the scientific advisory board of Ipsen. K.V.K. is a consultant for 89bio, CymaBay Therapeutics, Enanta Pharmaceuticals, Genfit, Gilead, HighTide Therapeutics, Inipharm, Intercept Pharmaceuticals, Madrigal, Mirum, NGM Biopharmaceuticals, Pfizer and Zydus Pharmaceuticals; and reports research support from 89bio, Boston, Corcept Therapeutics, CymaBay Therapeutics, Genfit, Gilead, GSK, Hanmi Pharmaceuticals, Intercept Pharmaceuticals, Janssen Pharmaceuticals, Madrigal, Mirum, NGM Biopharmaceuticals, Novo Nordisk, Pfizer, Pliant, Terns Pharmaceuticals, Viking Therapeutics and Zydus Pharmaceuticals; and reports speaker's fees from AbbVie, Gilead and Intercept Pharmaceuticals; and stock options in Inipharm. P.I. is in the scientific advisory board of Ipsen, Gilead, Zydus, Mirum, Advanz. M.C. has received speaker fees from Albireo/IPSEN, Advanz/Intercept, Amgen, GSK, Mirum Pharma, Gilead and advisory/consultancy fees from Advanz/Intercept, Alphasigma, Albireo/IPSEN, Mirum, GSK, Dr. Falk Pharma, Amgen, Mayoly, Echosens, Kowa, Moderna, Zydus. E.L.C. has received speaker fees from Albireo/IPSEN, Advanz/Intercept, Dr. Falk, Amgen, GSK, Mirum Pharma, Gilead, Chemomab, Zenus Bio and advisory/consultancy fees from Advanz/Intercept, IPSEN, Mirum, GSK, Dr. Falk Pharma, Amgen, Zenus Bio, Sanofi, Moderna and Sail BioPharma.

## Supporting information


**Figure S1:** Flowchart of patients' recruitment.
**Figure S2:** Subgroup analysis considering only PSC patients with (A) and without IBD (B) in the derivation cohort.
**Figure S3:** Distribution of the predicted probabilities of PSC diagnosis in the observed IRC patients in the validation cohort.
**Table S1:** Distribution of radiological pancreatic abnormalities identified in the derivation cohort.
**Table S2:** Estimated parameters of the model after excluding IRC patients.
**Table S3:** Discordant cases between expert diagnosis, non‐expert clinician assessment and PSC‐DM predictions.

## Data Availability

The data that support the findings of this study are available from the corresponding author upon reasonable request.
