# Supplementary material for: PSC‐DM: A Validated Clinical Diagnostic Model for Distinguishing Primary From Secondary Sclerosing Cholangitis
Source: Liver Int. 2026 Sep 30;46(11):e70892. doi: 10.1111/liv.70892 (PMC13625024; doi:10.1111/liv.70892)
Supplement: Supplementary file 1 [file LIV-46-0-s001.docx]

**Title: PSC-DM: a validated clinical diagnostic model for distinguishing primary from secondary sclerosing cholangitis**

**Short title: PSC-DM: a validated diagnostic model for PSC**

**Miki Scaravaglio*^1,2^, Rodrigo V. Motta*^3^**, Laura Cristoferi^4^, Cesare Maino^5^, Alberto Marini^1^, Eugenia Nofit^4^, Eugenia Pesatori^6^, Camilla Gallo^1^, Francesca Bolis^1^, Daphne D’Amato^1^, Armando Curto^7^, Federica Malinverno^4^, Alessio Gerussi^1,4^, Raffaella Viganò^8^, Mauro Viganò^6^, Stephen J. Rossi^9^, Kris V. Kowdley^10^, Pietro Invernizzi^1,4^, **Alessandra Nardi^†11^, Marco Carbone^†‡1,8^, Emma L. Culver^†3,12^**

* These authors share first authorship

† These authors share senior authorship

‡ Corresponding author

1. Department of Medicine and Surgery, University of Milano-Bicocca, Italy
2. Cambridge Stem Cell Institute, University of Cambridge, Cambridge, UK
3. Translational Gastroenterology and Liver Unit, John Radcliffe Hospital, and Nuffield Department of Medicine, University of Oxford, Oxford, UK
4. Division of Gastroenterology, Center for Autoimmune Liver Diseases, IRCCS Fondazione San Gerardo dei Tintori, ERN-RARE LIVER - Monza, Italy
5. Department of Diagnostic Radiology, Fondazione IRCCS San Gerardo dei Tintori, Monza, Italy
6. Hepatology Unit, ASST Papa Giovanni XXIII, Bergamo, Italy
7. Dipartimento di Medicina Sperimentale e Clinica, University of Florence, Largo Giovanni Alessandro Brambilla, 3, 50134 Firenze Italy
8. Hepatology and Gastroenterology Unit, ASST Grande Ospedale Metropolitano Niguarda, Milan, Italy
9. PSC Partners Seeking a Cure, Greenwood Village, Colorado, USA
10. Liver Institute Northwest and Elson S. Floyd College of Medicine, Washington State University, Seattle, Washington, USA
11. Department of Mathematics, University of Rome Tor Vergata, Rome, Italy
12. National Institute of Health Research (NIHR) Oxford Biomedical Research Center (BRC), University of Oxford, Oxford, UK

**Authors’ contributions**: MC, EC, AN, MS, RVM, LC have participated in the study concept and design. AN performed, MC and MS participated in data analysis. MS, RVM, AM, CM, EN, EP, CG, FB contributed to recruitment and/or ascertainment of participants. MS and RVM have verified the underlying data and wrote the first draft. All authors critically read and contributed to the final version of the manuscript.

**Correspondence to:**

Marco Carbone, MD, PhD

Department of Medicine and Surgery

University of Milano-Bicocca, Italy

[marco.carbone@unimib.it](mailto:marco.carbone@unimib.it)

**Table of contents**

Supplementary methods ……………………………………………………………………4

Supplementary tables ………………………………………………………………………6

Supplementary figures ………………………………………….…………………….........9

References …………………………………………………………………………………12

**Supplementary methods**

***Diagnostic definitions***

*Sclerosing cholangitis* was defined by the presence of characteristic intrahepatic and/or extrahepatic biliary strictures on MRCP or ERCP and/or histological evidence of periductal fibrosis.

According to EASL and AASLD guidelines^1,2^, *PSC* diagnosis required characteristic cholangiographic findings of multifocal strictures and segmental dilatations involving the intrahepatic and/or extrahepatic biliary tree, or compatible histological features in patients with normal cholangiography (small-duct PSC), with exclusion of secondary causes of sclerosing cholangitis (SSC). Patients with PSC-autoimmune hepatitis variant syndrome were included within the PSC group.

*Large duct PSC* was diagnosed in adult patients presenting with persistently elevated serum markers of cholestasis and typical findings of sclerosing cholangitis on high-quality cholangiography and after exclusion of secondary causes. The preferred diagnostic test was magnetic resonance cholangiopancreaticography (MRCP).

*Small duct PSC* was diagnosed in patients with persistently elevated serum markers of cholestasis of unknown cause, normal high-quality cholangiography, and compatible histology of PSC, particularly in those with concomitant inflammatory bowel disease (IBD).

*Typical PSC cholangiographic features* were defined as the presence of multifocal strictures and dilatations or ectasias involving the intra and/or extrahepatic biliary tree. Other typical findings include ductal thickening and pruning^3^.

*Histologic features compatible with PSC* included periductal fibrosis and fibro-obliterative cholangitis^4^.

*SSC* was diagnosed when a definite underlying aetiology for sclerosing cholangitis was identified, including biliary tract injury, ischaemic cholangiopathy, choledocholithiasis, chronic pancreatitis, genetic cholangiopathies, infectious cholangitis, toxic injury, or IgG4-related cholangitis (IRC)^5,6^. For patients with lithiasis associated SSC, classification required not only evidence of gallstone disease but also longitudinal clinical, biochemical, and radiological assessment supporting a causal relationship between biliary lithiasis and strictures. Specifically, all cases underwent a minimum follow-up of 12 months before final classification. The absence of chronic cholestasis and the stability or improvement of the biliary abnormalities after appropriate treatment of gallstone disease were considered supportive of SSC diagnosis rather than PSC with concomitant gallstone disease. *Low phospholipid-associated cholelithiasis (LPAC) syndrome* was considered in the presence of at least two out of the three following clinical features: symptomatic cholelithiasis before the age of 40 years, recurrence after cholecystectomy and/or evidence of intrahepatic lithiasis^7^. All patients with LPAC underwent genetic testing.

*IgG4-related cholangitis (IRC)* was diagnosed according to the HISORt criteria^8^, integrating histopathological (H), imaging (I), and serological (S) features including serum IgG4, other organ manifestations (O) of IgG4-RD and response to corticosteroid treatment where available (Rt)^8,9^.

**Supplementary tables**

**Supplementary Table S1. Distribution of radiological pancreatic abnormalities identified in the derivation cohort**

| **Pancreatic abnormalities** | **PSC (n=155)** | **SSC (n=79)** |
| --- | --- | --- |
| Pancreatic enlargement with loss of lobular contours | 0 (0.0%) | 5 (6.3%) |
| Pancreatic calcifications | 0 (0.0%) | 8 (10.1%) |
| Pancreatic duct irregularities | 2 (1.3%) | 12 (15.2%) |
| Pancreatic atrophy | 0 (0.0%) | 7 (8.9%) |
| Pancreatic cysts | 0 (0.0%) | 2 (2.5%) |

**Supplementary Table S2. Estimated parameters of the model after excluding IRC patients**

|  | **Parameter**  **estimate** | **Standard**  **error** | **LR Chi square** | **p value** |
| --- | --- | --- | --- | --- |
| Intercept | 6.5489 | 1.3924 |  |  |
| Age (years) | -0.1180 | 0.0257 | 39.8173 | <.0001 |
| IBD | 4.8866 | 1.4386 | 31.0614 | <.0001 |
| Previous abdominal surgery | -2.6039 | 0.6462 | 18.6434 | <.0001 |
| Autoimmune comorbidities | 2.0973 | 0.8167 | 7.0653 | 0.0079 |
| Familial autoimmunity | 2.2907 | 0.8867 | 6.4728 | 0.0110 |
| Pancreatic abnormalities | -2.4782 | 1.2431 | 4.1132 | 0.0426 |

**Supplementary Table S3. Discordant cases between expert diagnosis, non-expert clinician assessment and PSC-DM predictions.**

| **Patient id** | **Expert diagnosis** | **Non expert diagnosis** | **Cross validated probabilities of PSC estimated by PSC-DM** |
| --- | --- | --- | --- |
| 6 | PSC | SSC | **0.99915** |
| 20 | PSC | SSC | **0.99915** |
| 32 | PSC | SSC | **0.96963** |
| 43 | PSC | SSC | **0.99977** |
| 44 | PSC | SSC | **0.96696** |
| 52 | PSC | SSC | **0.96406** |
| 124 | PSC | SSC | **0.99671** |
| 125 | PSC | SSC | **0.89734** |
| 149 | SSC | PSC | 0.79312 |
| 165 | SSC | PSC | **0.35352** |
| 170 | SSC | PSC | 0.74306 |
| 180 | SSC | PSC | **0.12953** |
| 194 | PSC | SSC | **0.86780** |
| 222 | SSC | **SSC** | 0.92337 |
| 238 | PSC | SSC | **0.99963** |
| 239 | PSC | SSC | 0.10920 |
| **Notes:** In bold correctly classified cases relative to expert opinion. **Abbreviations:** PSC, primary sclerosing cholangitis; SSC, secondary sclerosing cholangitis. | | | |

**Supplementary figures**

**Supplementary Figure S1: Flowchart of patients’ recruitment**

**Supplementary Figure S2. Subgroup analysis considering only PSC patients with (A) and without IBD (B) in the derivation cohort.**

**A	B**

**Supplementary Figure S3: Distribution of the predicted probabilities of PSC diagnosis in the observed IRC patients in the validation cohort.**

**References**

1.	Chazouilleres O, Beuers U, Bergquist A, et al. EASL Clinical Practice Guidelines on sclerosing cholangitis. Journal of Hepatology. 2022;77(3):761-806. doi:10.1016/j.jhep.2022.05.011

2.	Bowlus CL, Arrivé L, Bergquist A, et al. AASLD Practice Guidance on Primary Sclerosing Cholangitis and Cholangiocarcinoma. Hepatology. Published online September 9, 2022:hep.32771. doi:10.1002/hep.32771

3.	Schramm C, Eaton J, Ringe KI, Venkatesh S, Yamamura J, for the MRI working group of the IPSCSG. Recommendations on the use of magnetic resonance imaging in PSC‐A position statement from the International PSC Study Group. Hepatology. 2017;66(5):1675-1688. doi:10.1002/hep.29293

4.	Ponsioen CY, Assis DN, Boberg KM, et al. Defining Primary Sclerosing Cholangitis: Results From an International Primary Sclerosing Cholangitis Study Group Consensus Process. Gastroenterology. 2021;161(6):1764-1775.e5. doi:10.1053/j.gastro.2021.07.046

5.	Gossard AA, Angulo P, Lindor KD. Secondary Sclerosing Cholangitis: A Comparison to Primary Sclerosing Cholangitis. Am J Gastroenterology. 2005;100(6):1330-1333. doi:10.1111/j.1572-0241.2005.41526.x

6.	Lazaridis KN. Sclerosing Cholangitis Epidemiology and Etiology. *Journal of Gastrointestinal Surgery*. 2008;12(3):417-419. doi:10.1007/s11605-007-0344-3

7.	Dong C, Condat B, Picon-Coste M, et al. Low-phospholipid-associated cholelithiasis syndrome: Prevalence, clinical features, and comorbidities. JHEP Rep. 2021;3(2):100201. doi:10.1016/j.jhepr.2020.100201

8.	Löhr JM, Beuers U, Vujasinovic M, et al. European Guideline on IgG4-related digestive disease – UEG and SGF evidence-based recommendations. United European Gastroenterology Journal. 2020;8(6):637-666. doi:10.1177/2050640620934911

9.	On W, Huggett MT. European Guideline on IgG4-related digestive disease: UEG and SGF evidence-based recommendations. *Frontline Gastroenterol*. 2022;13(2):171-174. doi:10.1136/flgastro-2021-101884
